# Using the RE‐AIM and TDF frameworks to evaluate the implementation of a standardized cognitive assessment protocol in outpatient rehabilitation

**DOI:** 10.1002/pmrj.13250

**Published:** 2024-08-19

**Authors:** Carla Tierney‐Hendricks, Megan E. Schliep, Minsi Sun, Perman Gochyyev, Christopher Carter

**Affiliations:** ^1^ Spaulding Rehabilitation Hospital Boston Massachusetts USA; ^2^ MGH Institute of Health Professions Boston Massachusetts USA

## Abstract

**Background:**

Impairments in cognition significantly affect patient functioning and rehabilitation outcomes. Assessment is essential to identifying at‐risk individuals and guiding care plans.

**Objective:**

A cognitive assessment protocol was implemented in occupational therapy (OT) and speech‐language pathology (SLP) outpatient practice. Using the Reach, Effectiveness, Adoption, Implementation, and Maintenance (RE‐AIM) framework and Theoretical Domains Framework (TDF), this study (1) measured the reach and adoption of the cognitive assessment protocol and (2) explored determinants and strategies that may affect adoption.

**Design:**

Sequential mixed methods.

**Setting:**

Two outpatient rehabilitation clinics (A and B) within a health care network.

**Participants:**

Medical records from 220 adult patients with neurologic diagnosis and 15 OT and SLP clinicians.

**Interventions:**

Cognitive assessment protocol.

**Main Outcome Measure(s):**

*Reach* of the assessment protocol across patient characteristics and *adoption* across clinical sites were measured quantitatively via retrospective electronic medical records review. Qualitative data on *effectiveness* and the *implementation* process were collected via clinician focus groups.

**Results:**

Protocol adoption rates were 71% and 54% at clinics A and B, respectively. Site B OT was more likely to be noncompliant with protocol adoption compared to Site A, when controlling for patient characteristics, (81% vs. 16%, respectively; odds ratio = 11.4, 95% confidence interval [3.36–38.64], *p* ≤ .001). Patient age was a significant factor for protocol reach; older age was associated with noncompliance of the SLP protocol adoption, *p* < .05. Both sites employed implementation strategies targeting the provider level (eg, education/training); Site A additionally included organization‐level strategies (eg, leadership engagement). In the absence of organization‐level strategies, OT and SLP clinicians at Site B identified barriers related to leadership support, resources, and workflow.

**Conclusions:**

Standardized practice protocols are feasible to implement within the rehabilitation setting, though multilevel implementation strategies may be needed to promote adoption. Aligning practices with the needs, values and priorities of the organization, providers, and patients and families is imperative.

## INTRODUCTION

Cognitive impairment affects 30%–60% of individuals receiving postacute care and may co‐occur across a variety of health conditions.^1^ Impairments in cognition are a particularly common sequalae following neurologic events or diagnoses, such as stroke, traumatic brain injury, or neurodegenerative disease. Cognitive impairments are associated with negative recovery and participation outcomes^1^, ^2^, ^3^; however, they may frequently go unidentified due to assessment practices.^1^, ^4^, ^5^ Rouch and colleagues (2023) found that only 38% of patients had documentation of cognitive screening by any therapy discipline in the postacute setting.^1^ Beyond being essential in identifying potential impairments and developing a plan of care, cognitive screening and assessment is a policy priority, with the 2014 Improving Medicare Post‐Acute Care Transformation Act calling for implementation of standardized data measures for cognitive status in multiple settings across the care continuum.^1^, ^6^, ^7^

The Cognitive Rehabilitation Manual (CRM), developed by the American Congress of Rehabilitation Medicine, published guidelines for cognitive rehabilitation assessment and intervention to promote the translation of evidence‐based recommendations into practice.^8^, ^9^ Assessment of cognition is included in the scope of practice for both occupational therapy (OT) and speech‐language pathology (SLP) clinicians.^10^, ^11^, ^12^ However, the adoption of these guidelines within routine clinical settings is unknown. The purpose of this manuscript is to describe a local quality improvement initiative aimed at standardizing cognitive assessment practices in the outpatient rehabilitation setting. Implementation science (IS) frameworks were used to evaluate adoption of the protocol across two sites, understand strategies supporting adoption, and examine facilitators and barriers to use of the cognitive assessment protocol.

### IS frameworks

**FIGURE 1:**
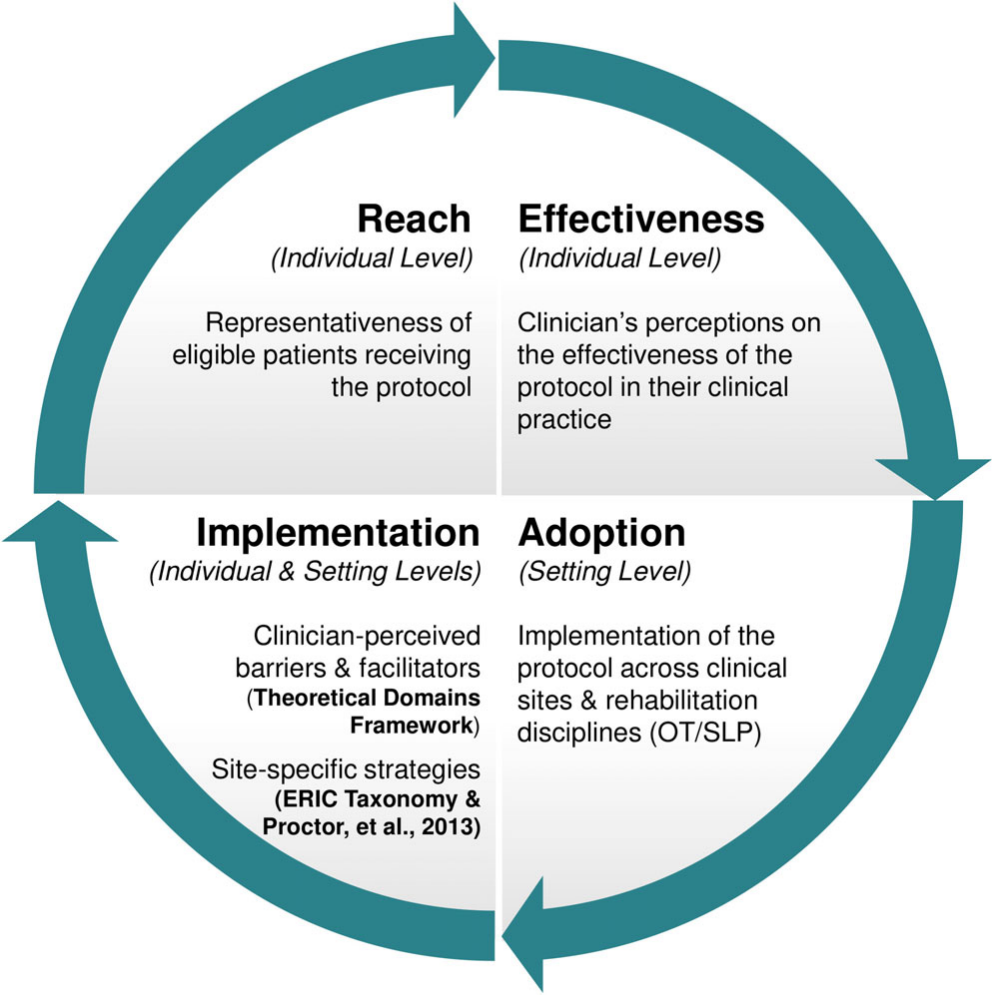
Application of the reach, effectiveness, adoption, implementation, and maintenance (RE‐AIM) framework domains of reach, effectiveness, adoption, and implementation within the current study. Within the Implementation domain, the Theoretical Domains Framework (TDF) was used to characterize barriers and facilitators of the implementation process. ERIC, Expert Recommendations for Implementing Change; OT, occupational therapy; SLP, speech‐language pathology.

IS is the study of methods to promote the uptake of evidence‐based practices (EBPs) into routine care to improve the quality and effectiveness of health services.^13^ Implementation frameworks offer a structure and common language to (1) guide or describe the process of implementation, (2) explore factors influencing implementation, and (3) evaluate the outcomes of implementation efforts.^14^, ^15^ Within the current study, the Reach, Effectiveness, Adoption, Implementation and Maintenance (RE‐AIM)^16^ framework was used as the overarching scaffold to evaluate the initial implementation of the cognitive assessment protocol and understand factors influencing implementation. The Theoretical Domains Framework (TDF)^17^, ^18^ was used to enhance our understanding of RE‐AIM's implementation domain, with a specific focus on identifying and describing implementation barriers and facilitators from the perspective of OT and SLP clinicians (Figure 1). Behavioral determinants identified using the TDF can guide the selection and design of behavior change strategies that address barriers and leverage facilitators.^19^

The aims of the current study were to (1) measure the adoption and reach of the cognitive assessment protocol at two outpatient clinics within the same rehabilitation network during a 3‐month period via a retrospective electronic medical record (EMR) review; and (2) explore determinants and strategies that may affect adoption, as well as clinicians' perceived effectiveness of the protocol via focus groups with OT and SLP clinicians. This was an initial exploratory study to guide future prospective implementation efforts.

## METHODS

**FIGURE 2:**
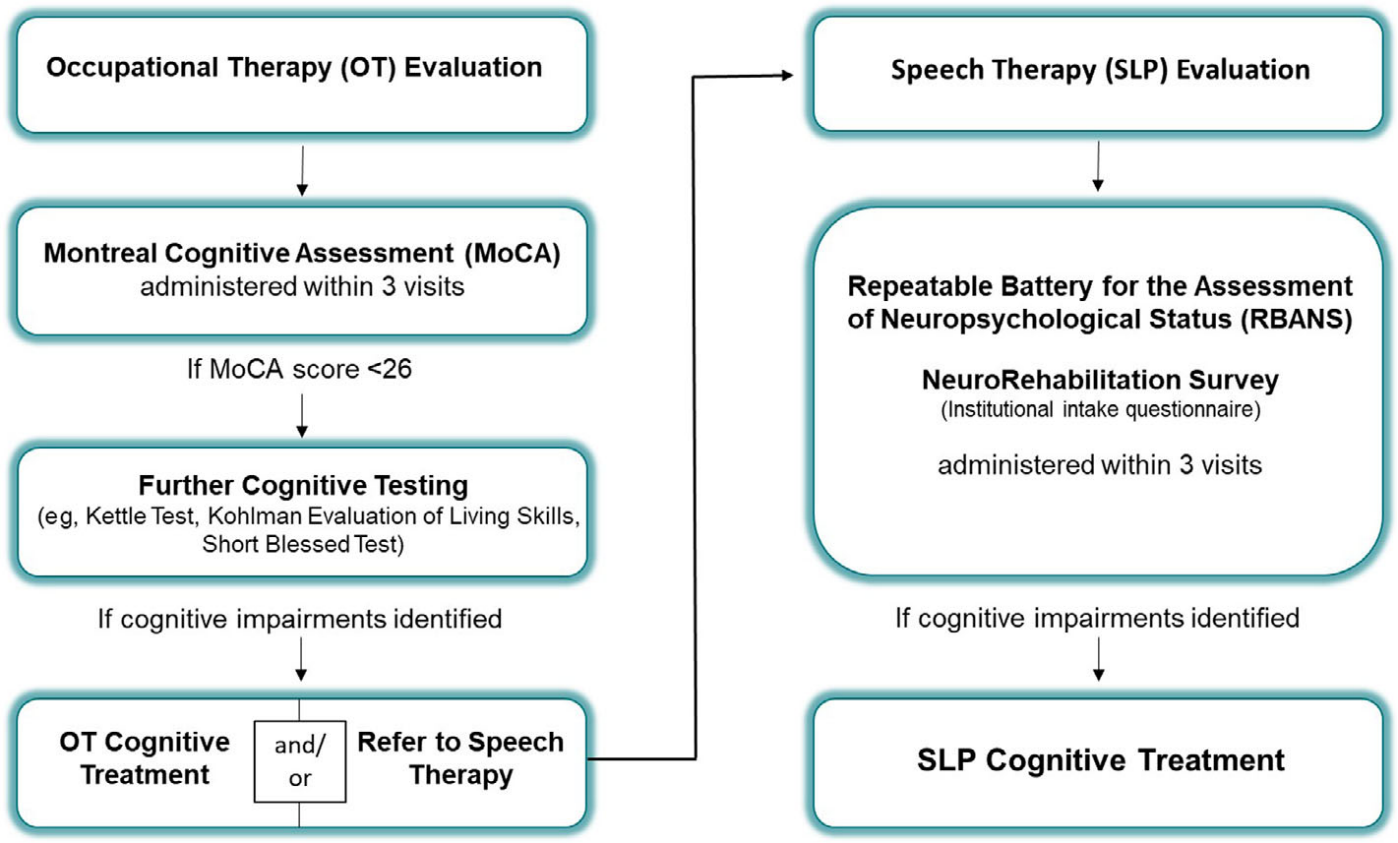
The cognitive assessment protocol for local implementation initiative.

The cognitive assessment protocol was developed through a quality improvement process. IS research methods were used to evaluate outcomes of this initiative and generate knowledge to inform future implementation work within the field of rehabilitation.^20^ In 2017, an interprofessional group of OT and SLP clinicians established a standardized assessment protocol for cognitive rehabilitation in outpatient care. Assessment measures were identified based on review and consensus from senior clinicians, who considered psychometric strength and clinical relevance and feasibility. The protocol (Figure 2), based on CRM guidelines and clinical expertise, offered an algorithm for cognitive assessment with an accompanying training and structure for documentation. The protocol was then implemented at two outpatient sites within a rehabilitation hospital network. Our research team partnered with the clinical team in 2020 to evaluate the initial implementation of the cognitive assessment protocol. The subsequent methods describe the research evaluation.

### Design

Guided by the RE‐AIM and TDF frameworks, this study was a post‐implementation evaluation and used a sequential mixed methods design. A retrospective medical record review was conducted to gather quantitative data on reach and adoption of the protocol at two outpatient clinics within a rehabilitation hospital network. We then gathered qualitative data to understand the effectiveness of the protocol on clinical decision‐making and explore ongoing barriers and facilitators. We also described planned and naturally occurring implementation strategies employed at each clinical site through retrospective evaluation of the implementation process. Data integration was completed using side‐by‐side comparison approaches.^21^ Both data sources were summarized within the discussion section.

### Participants

#### Clinical sites

Outpatient Site A is located in suburban Boston, with an annual neurological evaluation volume of approximately 110 and 145 patients for OT and SLP, respectively. Outpatient Site B is located within Boston, with an annual neurological evaluation volume of approximately 305 and 335 patients for OT and SLP, respectively.

#### Electronic medical record review

Eligible medical records included patients who met the following criteria: (1) 18 years of age or older, (2) presented for an evaluation with a neurologically based visit type (“Eval Neuro” or “Eval TBI”), and (3) seen by OT and/or SLP clinicians at one of the two outpatient clinics between August 2021 and October 2021. This time period was selected based on when our research team initiated our collaboration with the clinical team and to avoid workflow disruptions due to the pandemic.

#### Clinician focus groups

Eligible focus group participants included certified OT or SLP clinicians who worked in a full‐ or part‐time capacity within the two outpatient clinics and had received training in the protocol. Site A (suburban) had two OT and two SLP clinicians and Site B (urban) had seven OT and six SLP clinicians eligible for participation. Of note, focus groups were conducted with all eligible clinicians in March and April of 2022. Although many of these clinicians' charts were included in the August–October 2021 EMR review, this was not a criterion for focus group participation given expected changes in clinical staffing.

### Procedures

#### Reach and adoption: medical record review (quantitative)

For this study, *reach* was defined as the proportion and representativeness of patients with neurological diagnoses who received the protocol out of those who should have received the protocol based on diagnosis. Table 1 displays patient sociodemographic and diagnosis characteristics used to evaluate reach. *Adoption* of the protocol was defined as the administration of the cognitive screener/assessment(s) consistent with the protocol, and, if the patient's performance on the screener/assessment indicated risk of cognitive impairment, the provider documented (1) addressing cognition within OT and/or SLP plan of care, (2) recommending a referral to SLP services, or (3) documenting the rationale for deferring the evaluation of cognition (eg, patient with baseline cognitive deficits and living in a fully supported environment; SLP referral for specific motor speech and voice deficits). Records with lack of evidence that the protocol was considered and/or used in any of these ways was categorized as “noncompliant.”

**TABLE 1:** Patient characteristics.

	Total sample (*n* = 220)	Clinic A (*n* = 54)	Clinic B (*n* = 166)
Age (years), mean (SD)	56.3 (16.9)	61.7 (13.1)	54.6 (17.7)
Male gender: *n* (%)	114 (51.8%)	29 (53.7%)	85 (51.2%)
Race: *n* (%)			
Asian	16 (7.3%)	1 (1.9%)	15 (9.0%)
African American or Black	21 (9.5%)	‐	21 (12.7%)
White	155 (70.5%)	48 (88.9%)	107 (64.5%)
Other or unavailable^a^	28 (12.7%)	5 (9.3%)	23 (13.9%)
Ethnicity: *n* (%)			
Hispanic	17 (7.7%)	‐	17 (10.2%)
Non‐Hispanic	179 (81.4%)	42 (77.8%)	137 (82.5%)
Unavailable	24 (10.9%)	12 (22.2%)	12 (7.2%)
Primary language: *n* (%)			
English	201 (91.4%)	52 (96.3%)	149 (89.8%)
Other^b^	19 (8.6%)	2 (3.7%)	17 (10.2%)
Primary diagnosis: *n* (%)			
Cerebrovascular disease	77 (35.0%)	22 (40.7%)	55 (33.1%)
Concussion	25 (11.4%)	4 (7.4%)	21 (12.7%)
Neurologically based cancer/tumor	11 (5.0%)	1 (1.9%)	10 (6.0%)
Brain injury–traumatic and anoxic	27 (12.3%)	6 (11.1%)	21 (12.7%)
Neurodegenerative	28 (12.7%)	12 (22.2%)	16 (9.6%)
Congenital/developmental disorder	11 (5.0%)	1 (1.9%)	10 (6.0%)
Other^c^	41 (18.6%)	8 (14.8%)	33 (19.9%)
Premorbid employment status: *n* (%)			
Full‐ or part‐time/self‐employed/student	126 (57.3%)	21 (38.9%)	105 (63.3%)
Retired	58 (26.4%)	22 (40.7%)	36 (21.7%)
Disabled	25 (11.4%)	9 (16.7%)	16 (9.6%)
Other^d^	11 (5.0%)	2 (3.7%)	9 (5.4%)

*Note*: Dashes (−) indicate a zero value.

^a^ Other race: A race other than the three listed categories or unavailable/declined in the medical record.

^b^ Other primary language: Spanish, Portuguese, Cantonese, Mandarin.

^c^ Other diagnosis categories: infectious diseases, autoimmune diseases, functional neurologic disorder, spinal cord injury, etc.

^d^ Other premorbid employment status categories: homemaker, temporary leave, unemployed, unknown.

A project specific audit tool programed in REDCap^22^ was used for EMR data collection. This audit tool, developed by the research team with input from clinicians and hospital managers, included (2) patient demographics and diagnosis, (3) clinical site and whether OT and/or SLP services were received, and (4) adoption of cognitive assessment protocol (Appendix 1). The response options in the audit tool for patient variables were generated based on the clinical data available within the EMR in discrete fields and/or SLP and OT clinical notes.

To maximize interrater reliability of data extraction from the EMR, the first five records were audited collaboratively by the first three authors. One author (M.S.) then independently extracted data from the EMR using the audit tool for all eligible records. Two other authors (M.E.S., C.T.H.) independently audited 5% of randomly generated records (10% of total records). Interrater reliability was calculated between M.S. and M.E.S. or C.T.H. using percent agreement (92.5%). Consensus on discrepancies was reached through discussion between these 3 authors.

#### Implementation and effectiveness: focus groups (qualitative)

Given that this work describes early phase implementation, *effectiveness* is defined as the impact of an evidence‐based cognitive assessment protocol on clinicians' practices and decision‐making. With successful adoption of the protocol, we can then examine downstream outcomes at the patient level in future iterations of this work. The *implementation* domain of RE‐AIM guided our understanding of the implementation process and ongoing barriers and facilitators.

Qualitative focus group data were gathered to understand effectiveness of the cognitive assessment protocol and implementation determinants at the two clinical sites. Authors M.S., an SLP master's student, and C.T.H., a researcher and practicing SLP clinician, facilitated focus group discussions. Four focus groups were conducted with (1) OT clinicians (*n* = 2) and (2) SLP clinicians (*n* = 2) from Site A, and (3) OT clinicians (*n* = 3) and (4) SLP clinicians (*n* = 8) from Site B. Focus groups were scheduled for 1 hour each and conducted via videoconference. A focus group question guide was developed addressing topics related to general perceptions of cognitive assessment practices, perceived effectiveness of the protocol, as well as barriers and facilitators to implementing the protocol (see Appendix 2). The focus groups were video‐recorded and later transcribed using the Zoom transcription feature. All transcripts were checked for accuracy by author M.S.

#### Implementation strategies

Individual meetings were held with a clinical manager at Site A and the site champion from Site B to gather information about processes and methods used prior to and during the implementation of the protocol. This information provided data to retrospectively describe implementation strategies employed at the two clinical sites.

### Analysis

#### Reach and adoption: Medical record review (quantitative)

Descriptive statistics were used to summarize the adoption of the protocol, as the proportion of EMR records documenting the use or consideration of the protocol in OT and SLP practices across clinical Sites A and B. Patient sociodemographic and diagnosis information was also summarized by site. A chi‐square test examined the association between protocol adoption by each site. Reach of the protocol at the patient level was examined through a series of chi‐square tests, evaluating the association between adoption of the protocol and patient characteristics (gender, race, ethnicity, primary language, diagnosis category, and preinjury work status).^23^, ^24^ A univariate logistic regression was conducted to evaluate the association between protocol adoption and the continuous variable of patient age. An adaptive linear step‐up procedure^25^ was used to adjust for multiple comparisons. Informed by the univariate analyses, multivariate logistic regression models were used to evaluate the association between protocol adoption within OT and SLP practice and clinical site and patient sociodemographic characteristics, thus controlling for the shared variance across the predictor variables. Statistical analyses were completed using R studio.^26^

#### Implementation and effectiveness: focus groups (qualitative)

Interview transcripts were entered into NVivo 12^27^ for data organization and analysis. Analysis of the written transcripts was conducted by M.E.S. and C.T.H., who are both researchers and practicing SLPs in the inpatient rehabilitation setting. Interview data were analyzed using a content analysis approach.^28^ Efforts to improve trustworthiness of the data were addressed by (1) memoing and team debriefing meetings, (2) attention to discrepant and supporting evidence within the data, and (3) triangulation across coders.^29^

##### Implementation

To understand clinician‐perceived barriers and facilitators to implementing the protocol, focus group data related to the implementation process were deductively coded along the constructs of the TDF (see Appendix 3 for codebook). M.E.S. and C.T.H. independently completed line‐by‐line coding for each of the four focus group transcripts. The independently generated TDF codes were then merged into a single transcript document within NVivo to identify overlapping and nonoverlapping codes. Consensus on coding was reached by returning to the TDF codebook, sharing perspectives and insights into multi‐meanings, and by looking for consistent and discrepant evidence within the data.

##### Effectiveness

M.E.S. and C.T.H. completed a secondary analysis of the transcript data to examine clinicians' perceptions of the effectiveness of the protocol on their clinical decision‐making and the factors that contributed to these perceptions. Procedures for this secondary analysis followed those described previously. Codes were generated inductively, and themes were identified through a collaborative process of subsumption and polarization.

##### Implementation strategies

Based on data provided by clinical manager at Site A and the site champion from Site B, authors M.E.S. and C.T.H. named each implementation strategy using the Expert Recommendations for Implementing Change (ERIC) taxonomy^30^ as a guide. Each strategy was then further specified using the recommendation by Proctor and colleagues.^31^ The specification of the implementation strategies was shared with the clinical manager and site champion to verify accuracy.

## RESULTS

### Participants

A total of 260 OT and SLP records across the two clinical sites were eligible and were included in the analysis. Of the 260 records, 40 patients had been evaluated by both SLP and OT during the study time period, yielding 220 unique patient cases. Information on patient characteristics for the total sample and at each clinical site is shown in Table 1.

### Results of reach and adoption: medical record review (quantitative)

Descriptive statistics revealed an overall higher adoption rate at Site A (71%) compared to Site B (54%), which was driven by a relatively high adoption rate in both OT and SLP practice at Site A, compared to a high adoption rate in SLP only at Site B (Table 2).

**TABLE 2:** Descriptive statistics for adoption rate by clinical site and discipline.

Discipline	Clinic site		
	Percent adoption (proportion)		
	A	B	Discipline totals
OT	84% (21/25)	19% (20/106)	31% (41/131)
SLP	62% (21/34)	94% (89/95)	85% (110/129)
Site totals	71% (42/59)	54% (109/201)	

Abbreviations: OT, occupational therapy; SLP, speech‐language pathology.

As described in the methods, adoption rates were operationalized as a composite that included documentation of the cognitive screener/assessment(s) within the first three visits or a rationale for deferring evaluation, and, if the patient's performance on the screener/assessment indicated risk of cognitive impairment, the provider documented (1) addressing cognition within OT and/or SLP plan of care, or (2) recommending a referral to SLP services. For OT practice, the Montreal Cognitive Assessment (MoCA) screener was documented within the first three visits for 45 of 131 patients (34%). A documented rationale for deferring OT cognitive assessment was reported for 5 of the 131 patients (4%). Of the patients with identified cognitive deficits (33 of the 45), nine patients did not have a documented plan of care for the management of their cognitive deficits, making the cases noncompliant with the protocol. There were no referrals to SLP practice within this sample. This yielded 41 OT cases where the protocol was adopted.

For SLP practice, cognitive assessments consistent with the protocol were documented within the first three visits for 107 of 129 patients (83%). A documented rationale for deferring SLP cognitive assessment was reported for eight of the 129 patients (6%). Of the patients with identified cognitive deficits (96 of the 107), five patients did not have a documented plan of care for the management of their cognitive deficits, making the cases noncompliant with the protocol. This yielded 110 SLP cases where the protocol was adopted.

The results of univariate analyses examining the association of site and patient sociodemographic factors on the adoption of the cognitive assessment protocol are displayed in Table 3. Site was significantly associated with protocol adoption in OT, $\chi^{2}$ (1) = 28.55, *p* < .001 and SLP practices, $\chi^{2}$ (1) = 20.31, *p* < .001. Patient age was negatively associated with SLP protocol adoption but was not statistically significant. Patient sociodemographic factors such as gender, diagnosis category, primary spoken language, race, ethnicity, and preinjury work status were not significantly associated with the adoption of the protocol.

**TABLE 3:** Association between clinical site and patient sociodemographic factors on protocol adoption.

Variables	OT practice				SLP practice			
	Univariate test statistic	*p* value	95% CI	Adjusted *p* value^a^	Univariate test statistic	*p* value	95% CI	Adjusted *p* value^a^
Diagnosis category	χ^2^(6) = 8.61	.197	[2.203–14.150]	.218	χ^2^(6) = 14.44	.025*	[6.434–23.191]	.070
Age	*F*_(1,129)_ = 3.76, *R* ^2^ = .028, coef. = − 6.355	.055	[−12.843–.133]	.112	*F*_(1,127)_ = 6.72–*R* ^2^ = .05–coef. = 9.86	.011*	[2.334–17.387]	.053
Gender	χ^2^(1) = 2.11	.146	[.005–9.928]	.218	χ^2^(1) = .17	.677	[0.000–1.983]	.216
Primary language spoken	χ^2^(1) = .11	.742	[0–1.494]	.402	χ^2^(1) = .005	.944	[.000–.025]	.419
Race	χ^2^(3) = 1.14	.767	[.079–2.813]	.402	χ^2^(3) = 4.76	.190	[1.520–8.372]	.218
Ethnicity	χ^2^(1) = 2.37	.124	[.009–6.635]	.216	χ^2^(1) = .13	.719	[.000–1.260]	.402
Preinjury work status	χ^2^(3) = 9.66	.022*	[2.341–22.129]	.070	χ^2^(3) = 4.77	.189	[.281–12.409]	.218
Clinical site	χ^2^(1) = 28.55	.000***	[11.160–49.829]	.001**	χ^2^(1) = 20.31	.000***	[5.100–43.261]	.001**

Abbreviations: OT, occupational therapy; SLP, speech‐language pathology.

^a^ Correction for multiple comparisons using an adaptive linear step‐up procedure.

* Denotes *p* value <.5.

** Denotes *p* value <.01.

*** Denotes *p* value <.001.

Patient diagnosis, race, primary language, and preinjury work status were not included in the final multivariate models given unequal distribution across category levels. The results indicated that clinical site was significantly associated with adoption of OT and SLP cognitive assessment protocols, when controlling for patient factors of age, gender, and ethnicity. For OTs, noncompliance with protocol adoption was greater at Site B compared to Site A (81% vs. 16%, respectively). The odds of noncompliance with the OT assessment protocol at Site B increased by a factor of 11.4 compared to Site A, when controlling for patient sociodemographic factors, *p* < .001. For SLPs, noncompliance with protocol adoption was greater at Site A compared to Site B (38% vs. 6%, respectively). The odds of noncompliance with the SLP assessment protocol at Site B decreased by 94% compared to Site A, when controlling for patient sociodemographic factors, *p* < .001. Patient age was significantly associated with protocol adoption in SLP practice, when controlling for clinical site, gender, and ethnicity. For each 1‐year increase in age, the odds of noncompliance with the SLP protocol significantly increased by 6%, *p* < .05. Patient gender and ethnicity were not significantly associated with protocol adoption in OT and SLP practices (Table 4).

**TABLE 4:** Logistic regression models of clinic site and patient factors associated with cognitive assessment protocol adoption in OT and SLP practices.

Variables	OT practice			SLP practice		
	OR	95% CI	*p* value	OR	95% CI	*p* value
Intercept	4.48	[0.24–85.12]	.318	0.038	[0.001–2.30]	.119
Age	0.98	[0.95–1.00]	.054	1.07	[1.001–1.13]	.025*
Gender—male	0.59	[0.24–1.47]	.254	0.94	[0.26–3.44]	.925
Clinic site—B	11.4	[3.36–38.64]	.000***	0.062	[0.014–0.279]	.000***
Ethnicity—Non‐Hispanic	34	[0.04–2.96]	.329	0.21	[0.02–2.63]	.226
Observations	120			112		
Log likelihood	−60.88			−30.65		
Pseudo R^2^	0.19			0.27		

*Note*: Independent variable reference levels: gender = female; clinical site = A; ethnicity = Hispanic.

Abbreviations: OR, odds ratio; OT, occupational therapy; SLP, speech‐language pathology.

* Denotes *p* value <.05.

*** Denotes *p* value <.001.

Statistical power was assessed with a significance level of *α* = .05. In investigating the relationship between the adoption of OT protocol and site, our study demonstrated a high statistical power, exceeding 99%. Similarly, for the relationship between the adoption of SLP protocol and site, the statistical power was approximately 97%. These high power estimates indicate a robust ability to detect true significant relationships between the adoption practices of OT and SLP protocols and site.

### Results of implementation and effectiveness: focus groups (qualitative)

#### Implementation

**FIGURE 3:**
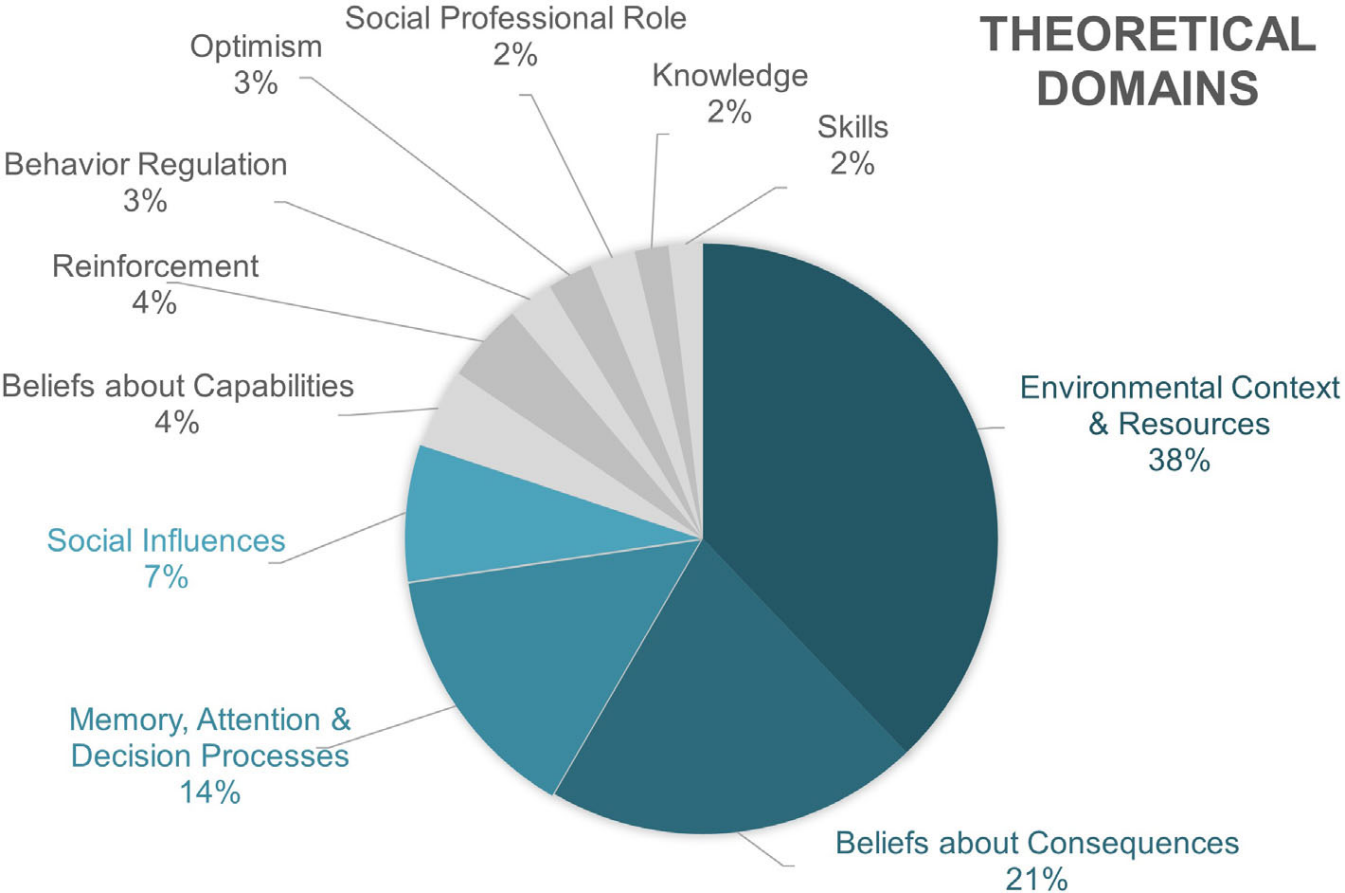
Distribution of qualitative codes related to the Theoretical Domain Framework (TDF) constructs. Each percentage represents the proportion of responses coded to a TDF category out of the total number of coded responses from the focus group data.

Figure 3 displays the distribution of codes aligning with the TDF constructs identified across the four focus group transcripts. Primary factors influencing clinicians' adoption of the protocol were related to the constructs of “environmental context and resources”; “beliefs about consequences”; “memory, attention, and decision processes”; and “social influences.” Table 5 displays supporting quotations.

**TABLE 5:** Qualitative results related to implementation determinants coded along the Theoretical Domains Framework (TDF).

Domain	Subcategory	Supporting quotation
Environmental context and resources	Physical environment	“In general we work out of a ridiculously crazy busy, very distracting gym, so that makes us trying to deliver cognitive services in general a real challenge.” (OT, Site B)
	Organizational support and leadership engagement	“It was kind of like so you're going to use this test, good luck!” (OT, Site B)
		“We didn't have comparable management involvement here, and we haven't, so when I think about it, I think that might have helped facilitate some of the goals of how implementing core competencies for cognition.” (SLP, Site B)
	Availability of assessment tools	“I mean we did this training like in May of 2021 or something, and then it took like 6 months or more with numerous prodding to get the assessments⋯.” (OT, Site B)
		“We still only [have] one copy of the RBANS…” (SLP, Site B)
	Workflow and productivity demands	“We're trying to get‐ trying to figure out if we can get the electronic scoring version of the RBANS [Repeatable Battery for Assessment of Neuropsychological Status] that's administered on iPads and scores automatically, because it takes a really long time to score the RBANS that we don't really have built into our day, and just in trying to figure all of the logistics⋯.” (SLP, Site B)
		“I like that it's a relatively quick test, you know, ideally we get 60 minutes for an eval, but sometimes it ends up only being 45, and it's nice that it can be done that quickly and still have time to do, you know, a quick case history, and if they haven't done the Neuro Survey yet I can usually get it all done in 45 minutes if I need to.” (SLP, Site A)
Belief about consequences	Standardization and data to support clinical decision‐making	“It's definitely a helpful framework like [clinician name] said. You know, to have the whole full complete, not only the whole, full, complete picture, but a score to go along with it.” (SLP, Site A)
		“If I hadn't had all that data and all those scores, maybe I would have been more nervous or hesitant to sort of call the doctors out. But I felt very confident in telling them an MRI should be done ⋯ So I think it can be kind of empowering that what the information that you get from it [the cognitive assessment] makes your decisions and your opinions stronger and easier.” (SLP, Site A)
		“For me I do think it has facilitated some referrals to speech therapy. Like if they're [clients] particularly having a difficult time with word finding in the fluency aspect. You know, and we didn't notice it right away but when I administered the MoCA [Montreal Cognitive Assessment], I noticed that those were very weak areas, and I've had a couple of clients that weren't initially scheduled for speech therapy, but I was able to refer them on because that was where their limitations were on the MoCA.” (OT, Site A)
	Standardization to support workflow	“It helps us be more efficient ⋯ it just minimizes the, you know, debate about which eval you should do to start.” (SLP, Site A)
		“Before we sort of started looking at this [using the protocol] ⋯ the Woodcock Johnson Revised or III, the WJ III tests of cognitive abilities that was kind of our go‐to. But there are 21 or 22 subtests, so you'd have to kind of pick and choose. You've got your memory ones, you got your nonverbal problem solving, and speed of processing, but that would certainly take least double or triple the time. So I'm very satisfied with—this is our decision tree now.” (SLP, Site B)
Memory, attention and decision processes	Automaticity in practice	“MoCA, a kind of general cognitive screening during the eval, if we run out of time during the eval, ⋯ then I'll make sure that's in my plan of care to do on first follow‐up, if not maybe second follow up. So it's always on my mind.” (OT, Site B)
		“I pretty much use the survey [NeuroRehab Survey] for all my neuro patients that come in.” (SLP, Site A)
	Flexibility within standard process	“I think the only thing I would say with that is just if the patient comes in with neuropsych testing, and they have so much information I'd be less likely to do the RBANS, because it's not going to give me as much information and more go off of just that interview with it, maybe the Neurorehab Survey and kind of finding out what the patient's goals are, rather than doing additional testing.” (SLP, Site B)
Social influences	Alignment with patient expectations	“If the patient's coming for their physical limitations, you know and they're just like I don't want to address that [cognition] ⋯ Then you have to kind of explain to them. Well, this is standard protocol. We have to do the assessment.” (OT, Site A)
	Alignment with Patient Needs	“So I would say that would be the one thing of just that individuality of some of our patients and what their needs are of whether I would jump right to testing or also, if they have testing fatigue, and want to just jump you know I don't need that information right away, and jump into some treatment for that buy‐in, and then do maybe testing at a later date.” (SLP, Site B)

*Note*: TEAL shading indicates facilitators to the domains. RED shading indicates challenges that influenced adoption of the protocol, which may also be interpreted as important considerations in standardizing clinical practices.

Abbreviations: OT, occupational therapy; SLP, speech‐language pathology.

##### Environmental context and resources

The domain of “environmental context and resources” includes aspects of the physical environment, organizational logistics, availability of resources and materials, and aspects of clinical workflow. OT clinicians at both sites cited the physical environment as a barrier to implementing the protocol in their clinical practice. Clinicians from both disciplines at Site B reported barriers related to organizational support, leadership engagement, and the availability of materials. The impact of the protocol on workflow and productivity was seen as both a barrier and facilitator.

##### Belief about consequences

The “belief about consequences” domain was identified as a factor facilitating the adoption of the protocol. This domain encompasses clinicians' beliefs and perceptions regarding expected outcomes or consequences of a behavior in a given situation. Clinicians described the benefits of the data generated from the standardized protocol in supporting their clinical decision‐making and workflow, which in turn reinforced their use of the protocol. Clinicians valued having standardization and concrete data to support their recommendations and referrals.

##### Memory, attention, and decision processes

Factors related to the domain of “memory, attention, and decision processes” were identified primarily as a facilitator, but clinicians also raised considerations that affected their use of the protocol. The “memory, attention, and decision processes” domain includes the clinician's attention and recall of information for a given practice and the process in reasoning through options within a clinical situation. Clinicians described how the protocol became a routine “automatic” part of their practice. Though clinicians appreciated the benefit of standardization, they also reported valuing flexibility in the protocol for clinical decision‐making.

##### Social influences

“Social influences” is defined as interpersonal processes that can influence the providers feelings and behaviors and includes relationships with colleagues, clients, and their caregivers. Within the current data, clinicians described how the alignment of the assessment protocol with patient expectations, goals, and needs can influence their use of the protocol. Clinicians acknowledged the importance of the patient perspective in clinical care.

#### Effectiveness

The implementation factors discussed previously are related to how effective clinicians perceived the protocol to be. The focus group data revealed several key themes regarding clinicians' perspectives on the effectiveness of the protocol for clinical processes and decision‐making. The topics included workflow efficiency, the clinical benefits of a standardized framework, having concrete data to guide decision‐making, and incorporating practices addressing cognition into routine care. These topics aligned with data highlighted within the “belief about consequence” and “memory, attention. and decision‐making processes” domains of the TDF summarized earlier. The following quotation exemplifies the perceived benefit of the protocol: “I definitely think it's [the cognitive assessment protocol] opened clinicians' eyes. You know, to other areas that you can maximize. … I think we're doing so much dual treatment with cognition now, and I think you know, everyone in general is, you know, we do see a large population of Parkinson's as well. So I think we're looking a lot more into cognition, because of the MoCA” (OT, Site B).

#### Implementation strategies

Implementation strategies by clinical site are reported in Table 6. After M.E.S. and C.T.H.'s initial specification, the clinical manager at site B provided correction and clarification on the dose and temporality of Strategy 1: Education.

**TABLE 6:** Specification of implementation strategies informed by Proctor et al.,^31^ framework.

Strategy	Description
**Strategy 1: education (Sites A and B)**	
Definition	Background knowledge to target users of an innovation
Actor	Interprofessional team from the cognitive rehabilitation task force
Action	Developed video‐recorded presentations regarding (1) cognitive rehabilitation, (2) cognitive assessment for SLPs, and (3) cognitive assessment for OTs, which contained specific information about the assessment protocol and were required education for clinicians
Target of action	OT and SLP clinicians
Temporality	Prior to implementation; ongoing as part of onboarding process for new staff
Dose	Three 1‐hour video‐recorded presentations
Implementation outcome	Adoption
Justification^a^	Provide knowledge of ACRM guidelines for evidence‐based cognitive assessment and the local assessment protocol
**Strategy 2: documentation templates (Sites and & B)**	
Definition	A standard form with a specified location to enter clinical data into the medical record
Actor	Templates developed by an interprofessional team from the cognitive rehabilitation task force
Action	Templates built within the electronic medical record system for consistent documentation of local protocol to support clinical workflow and documentation
Target of action	OT and SLP clinicians
Temporality	Developed prior to implementation; utilized by clinicians throughout implementation
Dose	Used by clinicians daily
Implementation outcome	Adoption, Effectiveness
Justification^a^	Supports documentation practices and workflow; supports clinical team's use of data for their clinical decision‐making
**Strategy 3: site champions (Sites A and B)**	
Definition	An individual who is an expert in the innovation who provides “on the ground” expertise within the clinical setting
Actor	Senior OT or SLP clinician who is a member of the cognitive‐rehabilitation task force
Action	Providing consultation and ongoing education to fellow clinicians within their discipline
Target of action	OT and SLP clinicians
Temporality	During implementation
Dose	As needed
Implementation outcome	Reach, adoption
Justification^a^	Providing ongoing support, education, and reminders can reinforce initial education and completion
**Strategy 4: audit and feedback (Site A only)**	
Definition	A summary of clinical performance over a specified period that can be provided in written, electronic, or verbal format
Actor	Rehabilitation manager
Action	Using the REDCap audit tool to review clinical documentation and conduct one‐on‐one meetings with OT and SLP clinicians
Target of action	OT and SLP clinicians
Temporality	During implementation
Dose	Every 2–4 weeks
Implementation outcome	Reach, adoption
Justification^a^	Supports real‐time education and support during implementation; promotes optimism and buy‐in, provides opportunity to identify and address barriers
**Strategy 5: leadership engagement (Site A only)**	
Definition	Active involvement and cultivating an environment of support and value related to a clinical innovation
Actor	Rehabilitation manager
Action	Providing leadership support through regular check‐ins and discussions in department meetings
Target of action	OT and SLP clinicians
Temporality	Prior to and during implementation
Dose	Check‐ins every 2–4 weeks; Monthly department meetings
Implementation outcome	Reach, adoption, effectiveness
Justification^a^	Demonstrates perceived value by the organization for use of the protocol; promotes optimism and buy‐in, provides opportunity to identify and address barriers

Abbreviations: ACRM, American Congress of Rehabilitation Medicine; OT, occupational therapy; SLP, speech‐language pathology.

^a^ Given that strategies were implemented by the clinical team and were mapped retrospectively, the justification for each strategy reflects “pragmatic justification” based on the clinical team's decision‐making for the strategy.

## DISCUSSION

The current study used the RE‐AIM and TDF frameworks to (1) evaluate the reach and adoption of a standardized cognitive assessment protocol within OT and SLP practices across two outpatient sites and (2) explore determinants and strategies that support implementation of standardized metrics within the rehabilitation context. Our results revealed that implementation of a standardized cognitive assessment protocol is feasible within the outpatient setting with 54% and 71% adoption rates of the protocol at Site B and Site A, respectively. The adoption rates in the current study are consistent with rates in prior studies that examine standardized assessment practices in rehabilitation (40%–71%).^32^, ^33^, ^34^ Yet, despite this evidence for preliminary feasibility, differences in adoption rates of the cognitive assessment protocol were observed across clinical sites and disciplines. Adoption rates at Site A were relatively high and consistent across OT and SLP practices. Adoption rates in OT practices at Site B (19%) were significantly lower than the rates in OT practice at Site A (84%). The high adoption rates in SLP practice at Site B (94%) were observed and may be related to the assessment measures in the protocol being well established in practice prior to implementation.

Our evaluation of the implementation process sheds further light on factors that may have influenced differences in adoption rates. Both clinics employed strategies such as education/training, documentation templates, and site champions to support behavior change at the individual provider level. Interestingly, Site A, which had greater overall adoption of the protocol, also employed organizational‐level strategies including leadership engagement and auditing feedback. Without the support of organizational‐level strategies, clinicians at Site B identified barriers related to leadership support, availability of resources, and priority and workflow demands.

Poor organizational logistics, lack of resources, and time constraints are frequently cited within the rehabilitation literature as barriers to routine outcome measurement practices.^35^, ^36^, ^37^, ^38^ Though organizational‐level challenges are commonly identified, a majority of implementation studies in the field of rehabilitation have focused on individual‐level strategies, such as education and training.^39^, ^40^, ^41^ To promote successful and sustainable practice changes, it is critical to align implementation strategies to identified barriers.^42^, ^43^ There is a critical need to evaluate organizational context in the implementation of rehabilitation programs and protocols.^44^ The current work provides preliminary evidence to guide the design and testing of multilevel strategies at both the individual and organizational levels to promote practice change in the field of rehabilitation.

Clinicians identified factors related to “belief about consequences” and “memory, attention, and decision‐making” domains as facilitators to adopting the cognitive assessment protocol, which contributed to their perceptions of the effectiveness of the protocol to their clinical practice. Clinicians valued having a standardized approach and concrete data to guide their care plans and recommendations, as well as support workflow efficiency. The perceived value of an EBP has been shown in prior literature as a facilitator for the adoption of EBPs within speech, occupational, and physical therapy practices.^35^, ^36^, ^38^, ^45^, ^46^, ^47^ Having clear processes and systems for integrating assessment data into clinical decision‐making and care plans can contribute to clinicians' perceptions of the value of outcome measures.^48^, ^49^

Overall, patient factors, other than age, did not affect the reach of the protocol. As we identified, older patients were less likely to receive the protocol. Glenny and colleagues explored clinicians' perceptions on their use of standardized outcome measures with older adults, highlighting challenges related to instrument sensitivity and interpretation of scores given other comorbidities.^50^ A variety of patient‐specific factors were discussed by clinicians in focus groups as reasons they may defer or vary the protocol for a particular patient. Although age, specifically, was not mentioned, the alignment of a measure with patients' needs or status was linked to clinicians' perceptions on the value of the assessment measures. This is consistent with clinician perceptions of the value of clinical practices and their impact on adoption.^45^

## LIMITATIONS

Although this study added valuable insights into implementation processes to support the adoption of evidence‐based cognitive rehabilitation practices in real‐world care, there are limitations to acknowledge. First, this was an analysis of implementation efforts that had been initiated prior to the current evaluation. As such, implementation strategies at each site were identified and mapped retrospectively. Although this study provided preliminary evidence for the selection and design of implementation strategies, a prospective implementation study is needed to further evaluate the direct impact of these strategies on practice change. Secondly, we examined implementation only over a 3‐month time frame and thus did not explore the RE‐AIM domain of maintenance, which would be important to explore in the future iterations of our work. Additionally, three of four focus groups lasted for the fully scheduled 60 minutes; however, the focus group with OT clinicians at Site B was only 15 minutes due to limited clinician availability. All questions were presented by focus group facilitators, but responses may have been less robust. The number of clinical practitioners and patient volume were different between Site A and Site B, which may have had an impact on implementation efforts and uptake. Within a smaller clinic, it may be easier to build consensus, monitor practices and provide opportunities for discussion between leadership and clinicians. Finally, these sites were both within the same health care network; therefore, consideration must be taken when generalizing findings across institutions that are not part of a network or that vary in other setting characteristics.

## CONCLUSION

The implementation initiative within the current study was developed entirely by a team of invested clinicians and leadership partners, highlighting the relevance of this work to real world practice. Standardization of cognitive assessment practices within the rehabilitation setting is feasible and has the potential to directly impact clinician decision‐making and workflow. However, in establishing these protocols, it is necessary to align practices with the needs, resources, values, and priorities of the organization, the individual providers, and the patients and families who are receiving care. Multilevel implementation strategies may be needed to support adoption of standardized practices. Future research can build upon the current work by prospectively testing strategies targeting barriers at these levels.

## FUNDING INFORMATION

No external funding supported this work.

## DISCLOSURES

The authors report no conflicts of interest.

## Supporting information

Appendix 1.

Appendix 2.

Appendix 3.
