# Supplementary material for: Using the RE‐AIM and TDF frameworks to evaluate the implementation of a standardized cognitive assessment protocol in outpatient rehabilitation
Source: PM R. 2024 Aug 19;17(Suppl 2):S132–45. doi: 10.1002/pmrj.13250 (PMC12659721; doi:10.1002/pmrj.13250)
Supplement: Supplementary file 1 — Appendix 1. [file PMRJ-17-S132-s001.pdf]

Cognitive Assessment and Intervention Audit

Please complete the survey below.

Thank you!

Identifying Information

Auditor

____
(Last Name, First Name)

Audit Date

____

Clinician

____
(Last Name, First Name)

Discipline

◯ OT
◯ SLP
(Select One)

Clinic

◯ Braintree
◯ Brighton
◯ Cambridge
◯ Cape Ann
◯ Charlestown
◯ Downtown Crossing
◯ Foxborough
◯ Framingham
◯ Hanover
◯ Hyannis
◯ Lexington
◯ Lynne
◯ Malden
◯ Marblehead JCC
◯ Marblehead YMCA
◯ Marlborough
◯ Medford
◯ Orleans
◯ Peabody
◯ Plymouth
◯ Quincy
◯ Salem
◯ Sandwich
◯ Sandwich Pedi
◯ Wellesley
(Select One From Drop Down)

MRN#

____
(Include Numbers Only)

General Clinical Information

Patient Diagnosis	◯ Cancer<br>◯ Concussion<br>◯ Cerebrovascular Disease<br>◯ Congenital/Developmental<br>◯ Degenerative Conditions<br>◯ Dementia<br>◯ TBI<br>◯ Anoxic Brain Injury<br>◯ Tumor<br>◯ Other
Specific Dx/Stroke Location	__________________________________<br>(Please be as specific as possible utilizing physician orders, therapy notes, etc.)
Patient Age	__________________________________
Patient Sex	◯ Female<br>◯ Male<br>◯ Unknown<br>◯ X
Patient Primary Language	◯ Cantonese<br>◯ Cape Verdean Creole<br>◯ English<br>◯ Haitian Creole<br>◯ Mandarin<br>◯ Portuguese-Brazilian<br>◯ Russian<br>◯ Spanish<br>◯ Vietnamese<br>◯ Other<br>◯ Unavailable
Patient Race	◯ American Indian or Alaska Native<br>◯ Asian<br>◯ Black or African American<br>◯ Declined<br>◯ Native Hawaiian or Other Pacific Islander<br>◯ Other<br>◯ Unavailable<br>◯ White
Patient Ethnicity	◯ Hispanic<br>◯ Non-Hispanic<br>◯ Unavailable<br>◯ Other

Work Status	◯ Working Full-Time<br>◯ Working Part-Time<br>◯ Retired<br>◯ Unemployed<br>◯ Disabled<br>◯ Current Student<br>◯ Homemaker<br>◯ Military<br>◯ Self-Employed<br>◯ Temporary Leave<br>◯ Volunteer<br>◯ Other<br>◯ Unknown
Evaluation Date	______________________

OT Cognitive Assessment

MOCA performed by OT within 3 visits?	◯ Yes<br>◯ No
At which visit number was the MOCA performed?	◯ 1<br>◯ 2<br>◯ 3
MOCA Score	◯ 1<br>◯ 2<br>◯ 3<br>◯ 4<br>◯ 5<br>◯ 6<br>◯ 7<br>◯ 8<br>◯ 9<br>◯ 10<br>◯ 11<br>◯ 12<br>◯ 13<br>◯ 14<br>◯ 15<br>◯ 16<br>◯ 17<br>◯ 18<br>◯ 19<br>◯ 20<br>◯ 21<br>◯ 22<br>◯ 23<br>◯ 24<br>◯ 25<br>◯ 26<br>◯ 27<br>◯ 28<br>◯ 29<br>◯ 30

Cognitive Impairment Level Based on MoCA Score	◯ 26-30 Normal: No further evaluation of cognitive skills or cognitive rehabilitation required<br>◯ 19-25 Mild Impairment: Further evaluation of cognitive skills and cognitive rehabilitation required<br>◯ 10-18 Moderate Impairment: Further evaluation of cognitive skills and cognitive rehabilitation required<br>◯ 0-10 Severe Impairment: Further evaluation of cognitive skills and cognitive rehabilitation required
Were additional cognitive assessments performed by the OT?	◯ Yes<br>◯ No
Additional OT Cognitive Assessments Performed	☐ Clock Drawing Test<br>☐ Kettle Test<br>☐ Short Blessed Test<br>☐ Neuro Rehabilitative Survey<br>☐ Executive Function performance Test<br>☐ Kohlman Evaluation of Living Skills<br>☐ Rivermead Behavioral Memory Test<br>☐ Behavioral Inattention Test<br>☐ Motor-Free Visual Perception Test-4<br>☐ Other<br>(Select All That Apply)
Alternate Cognitive Assessment	______________________________
Planned cognitive intervention	☐ Patient is already receiving SLP services<br>☐ A referral to SLP services has been newly recommended in the Plan-of-Care<br>☐ Cognitive deficits will be managed within OT Plan-of-Care<br>☐ Based on screening/testing cognitive intervention indicated but not included in the plan of care<br>☐ Based on screening/testing cognitive intervention not indicated
If no formal cognitive screen or assessment was performed justification was documented in one or more of the following locations?	☐ Barriers To Learning Section of Progress Note<br>☐ Cognitive Function Flow Sheet<br>☐ Other<br>☐ Not Documented

SLP Cognitive Assessment

Were cognitive assessments performed within 3 visits by the SLP?	◯ Yes<br>◯ No
Cognitive assessments performed by the SLP	☐ RBANS<br>☐ CLQT<br>☐ APT<br>☐ FAVRES<br>☐ Neurorehabilitation Survey<br>☐ Rivermead Behavioral Memory Test<br>☐ Woodcock Johnson III<br>☐ Other

Alternate Cognitive Assessment	______________________________
Planned cognitive intervention	☐ Cognitive deficits will be managed within SLP Plan-of-Care<br>☐ Based on screening/testing cognitive intervention indicated but not included in the plan of care<br>☐ Based on screening/testing cognitive intervention not indicated
If no formal cognitive screen or assessment was performed justification was documented in one or more of the following locations?	☐ Barriers To Learning Section of the Progress Note<br>☐ Cognitive Function Flow Sheet<br>☐ Other<br>☐ Not Documented

Epic Specific

Epic Cognitive Flow Sheets Utilized?	◯ Yes<br>◯ No
Cognitive Smartphrase Utilized?	◯ Yes<br>◯ No

Comments

Comments	___________________________________<br>(Free Text)
