# Supplementary material for: Using the RE‐AIM and TDF frameworks to evaluate the implementation of a standardized cognitive assessment protocol in outpatient rehabilitation
Source: PM R. 2024 Aug 19;17(Suppl 2):S132–45. doi: 10.1002/pmrj.13250 (PMC12659721; doi:10.1002/pmrj.13250)
Supplement: Supplementary file 2 — Appendix 2. [file PMRJ-17-S132-s003.docx]

**Appendix 2:** Focus group question guide

| Topic Category | Question prompts |
| --- | --- |
| **General perceptions and practices** | - Overall, describe you experience assessing cognition when working with patients in the clinic. |
| **Experience and Perceptions regarding the Cognitive Assessment Protocol** | - Describe your experience using the Cognitive Assessment Protocol when assessing cognition with patients. - Tell me about a time when using the Protocol was helpful.   - Describe the specific aspect of the protocol? Patient population? Clinical situation? - Tell me about a time when using the Protocol was less helpful.   - Describe the specific aspect of the protocol? Patient population? Clinical situation? - Describe how the Protocol has impacted your decision-making or patient care in any way. - In your opinion, what would an ideal Cognitive Assessment Protocol look like? |
| **Implementation Process related to the Cognitive Assessment Protocol** | - What factors help you to implement the Protocol? - What barriers or challenges do you face in implementing the Protocol? - Describe any adaptations and adjustments made at your site in the process of implementing the Cognitive Assessment Protocol. |
